# *NFAT5* is differentially expressed in Sprague-Dawley rat tissues in response to high salt and high fructose diets

**DOI:** 10.1590/1678-4685-GMB-2018-0120

**Published:** 2019-02-28

**Authors:** Braden A. Herman, Kaylee M. Ferguson, Jared V.B. Fernandez, Samantha Kauffman, Jason T. Spicher, Rachel J. King, Julia A. Halterman

**Affiliations:** 1 Department of Biology, Eastern Mennonite University, Harrisonburg, VA, USA; 2 Master’s in Biomedicine Program, Eastern Mennonite University, Harrisonburg, VA, USA

**Keywords:** NFAT5, aldose reductase, salt, fructose, real-time PCR

## Abstract

Current diets contain an increasing amount of salt and high fructose corn syrup, but it remains unclear as to how dietary salt and fructose affect organ function at the molecular level. This study aimed to test the hypothesis that consumption of high salt and fructose diets would increase tissue-specific expression of two critical osmotically-regulated genes, nuclear factor of activated T-cells 5 (*NFAT5*) and aldose reductase (*AR*). Fifty Sprague-Dawley rats were placed on a control, 4% NaCl, 8% NaCl, or 64% fructose diet for eight weeks. Fourteen different tissue samples were harvested and snap-frozen, followed by RNA purification, cDNA synthesis, and *NFAT5* and *AR* gene expression quantification by real-time PCR.Our findings demonstrate that *NFAT5* and *AR* expression are up-regulated in the kidney medulla, liver, brain, and adipose tissue following consumption of a high salt diet. *NFAT5* expression is also up-regulated in the kidney cortex following consumption of a 64% fructose diet. These findings highlight the kidney medulla, liver, brain, and adipose tissue as being “salt-responsive” tissues and reveal that a high fructose diet can lead to enhanced *NFAT5* expression in the kidney cortex. Further characterization of signaling mechanisms involved could help elucidate how these diets affect organ function long term.

## Introduction

Cardiovascular disease is the number one cause of death in the United States, and over 35% of Americans have some form of cardiovascular disease, such as hypertension, atherosclerosis, coronary artery disease, heart attack, or stroke (Lloyd-Jones *et al.*, 2010). Similarly, 35% of individuals in the United States suffer from metabolic syndrome, which is characterized by elevated blood pressure and sugar levels, unhealthy levels of cholesterol, and excess fat around the waist, which increases the risk of complications (Aguilar *et al.*, 2015). Although clinical treatments for these conditions exist, they primarily serve as retroactive procedures to treat the disease and prevent further exacerbation of the disease, rather than proactive measures to prevent the development of disease. Diet is known to play a large role in promoting cardiovascular disease and metabolic syndrome through the alteration of gene expression within the body, which can lead to elevated blood pressure, high blood sugar, inflammatory sequelae, and dysfunction within various organ systems (He *et al.*, 1999; Johnson *et al.*, 2007). Evidence suggests that sustained consumption of a high salt and/or high fructose diet increases the risk and prevalence of hypertension and metabolic syndrome, thereby increasing the risk of heart disease, diabetes, and stroke (Graudal *et al.*, 2011).

In the current climate of strong debate over the role of salt in affecting one’s risk for developing hypertension, and the lack of information regarding how increased fructose consumption can affect the body, we sought to examine how a high salt and high fructose diet can alter gene expression. Nuclear factor of activated T-cells 5 (*NFAT5*) and aldose reductase (*AR*) gene expression have been extensively studied under high salt concentrations *in vitro*; however, it is unknown how high dietary salt consumption alters tissue-specific expression of NFAT5 and AR *in vivo*. Similarly, it is unknown how high dietary consumption of fructose affects *NFAT5* and *AR* gene expression *in vivo*. We hypothesized that consumption of a high salt and/or high fructose diet would enhance NFAT5 and AR expression in various organs, such as in the kidney and the liver. In this study, we present the first tissue-wide characterization of *NFAT5* and *AR* gene expression patterns following consumption of increased salt and/or fructose diets.

Salt is essential in the human diet, as it prevents muscle cramping and aids in water retention and nutrient absorption. Due to these necessary functions, salt has been a sought-after commodity for centuries. However, the overuse of salt in the human diet in recent decades has been linked with health problems affecting modern societies. One meta-analysis of 13 different studies of salt intake in roughly 170,000 individuals living in both developed and less developed regions of the world found a strong link between high salt diets and the recent increase in stroke and cardiovascular disease in humans (Strazzullo *et al.*, 2009). A study published in the New England Journal of Medicine used a projected model to determine the outcome on human health due to a decrease in dietary salt intake. The authors found that a novel decrease in the amount of salt in the human diet would yield a substantial decrease in cardiovascular disease, death from cardiovascular disease, and in turn, lower healthcare costs (Bibbins-Domingo *et al.*, 2010). Additionally, Sacks *et al.* (2001) found that implementation of a Dietary Approach to Stop Hypertension (DASH) low-salt diet was beneficial in lowering blood pressure, subsequently decreasing the risk of cardiovascular disease in the 412 study participants.

Contradictive to these studies, others indicate that increased salt consumption is tied to lower incidence of hypertension, while decreased salt consumption is correlated with higher incidence of hypertension (Moore, 2017). Although salt is required in the human diet, it is still unclear if its overconsumption is contributing to the epidemic of cardiovascular disease in the United States. Evidence indicates that sodium consumption alone is not the key factor driving hypertension development, but rather that hypertension is generated by an imbalance in sodium, potassium, magnesium, calcium, and chloride in one’s diet (Perez and Chang, 2014). However, salt-sensitivity, being genetically predisposed to health effects due to salt intake, is an additional factor that can put individuals at a higher risk for developing hypertension and cardiovascular disease (Burnier *et al.*, 2015).

Unlike salt, increased fructose consumption is a relatively recent change in the human diet, partly due to the addition of high fructose corn syrup in many processed foods. Recent clinical research has shown that high levels of fructose in the diet can be a key factor in developing attributes of metabolic syndrome (Johnson *et al.*, 2007). This could be due to fructose’s ability to lower the basal metabolic rate, and the unique step in fructose processing that results in an increased concentration of uric acid, a compound that has been shown to increase the risk of cardiovascular disease (Johnson *et al.*, 2007). Fructose is metabolized by fructokinase, an enzyme that contains no negative feedback system. The use of ATP in the unregulated phosphorylation cascade of fructokinase may result in ATP depletion, which can affect the transport of molecules through the cell membrane and cellular signaling that uses ATP to function. Fructose consumption has regularly been linked with an increased incidence of diabetes, as the lack of regulation of fructokinase in fructose metabolism can result in insulin resistance (Khitan and Kim, 2013).

NFAT5 (additionally referred to as tonicity responsive enhancer binding protein, TonEBP) is the only known mammalian transcription factor sensitive to changes in salt (NaCl) concentrations (Go *et al.*, 2004). NFAT5 is ubiquitously expressed throughout the body and has been shown to play a critical role in modulating cellular function in response to osmotic stress and disease (Lopez-Rodriguez *et al.*, 1999; Miyakawa *et al.*, 1999). Hypertonic (high salt) stress results in the phosphorylation of the C-terminal end of the NFAT5 transactivation domain and initiates the translocation of the NFAT5 protein from the cytoplasm of the cell to the nucleus (Lopez-Rodriguez *et al.*, 1999; Miyakawa *et al.*, 1999; Dahl *et al.*, 2001). NFAT5 subsequently binds to the promoter region of target genes responsible for osmoregulation, such as AR, sodium/myoinositol cotransporter, betaine transporter, and the sodium chloride/taurine cotransporter. Increased expression of these genes results in the gradual intracellular accumulation of osmolytes such as sorbitol, inositol, betaine, and taurine in order to restore the cell to an isosmotic environment (Miyakawa *et al.*, 1999; Ho, 2006; Zhang *et al.*, 2003; Lee *et al.*, 2011). Therefore, NFAT5 is a key transcription factor in the regulation of cellular homeostasis in response to high salt induced hypertonic stress (Ho, 2006). *AR*, a gene activated by the binding of NFAT5 to its promoter, is also a critical component of the mammalian response to hypertonicity-induced osmotic stress. AR catalyzes the creation of compatible organic osmolytes within the cell by converting glucose to sorbitol. Sorbitol is an organic osmolyte linked with the prevention of hypertonic stress-induced renal cell damage, thereby highlighting the role of AR in regulating the kidney’s adaptation to hypertonic stress (Ferraris *et al.*, 1994). Due to increased expression in response to salt-induced changes in tonicity, *NFAT5* and *AR* can therefore be considered “salt-responsive” genes (Lopez-Rodriguez *et al.*, 1999; Lee *et al.*, 2011; Luo *et al.*, 2016).

Although fructose is now a common nutrient in many individuals’ diets, no *in vitro* or *in vivo* studies have been conducted to determine if fructose can alter NFAT5 expression. As highlighted previously, NFAT5 expression and activation can be induced by tonicity-dependent mechanisms through changes in extracellular salt concentrations; however, NFAT5 expression can also be regulated via tonicity-independent mechanisms such as T-cell receptor activation, TGF-β, IL-1β, PDGF-BB, and TNF-α stimulation (Halterman *et al.*, 2012). Therefore, although untested, fructose could serve as a tonicity-dependent or tonicity-independent stimulator of NFAT5 expression*.*

## Materials and Methods

### Implementation of diet, blood pressure measurements, and tissue harvesting

Animal experiments were approved by Eastern Mennonite University’s Animal Care and Use Committee. Fifty male Sprague-Dawley rats (Charles River Laboratories, Malvern, PA) were used in this study. Only male rats were used in this study in order to decrease variability due to gender. Rats were 8 weeks old upon arrival and were all fed a control diet for one week while undergoing an acclimatization period with the CODA tail cuff blood pressure device (Kent Scientific, Torrington, CT).

Following acclimatization, rats were placed on one of four Teklad custom research diets (Harlan Laboratories, Frederick, MD) for 8 weeks. Fourteen rats were fed a control diet of 6% fructose and 0.25% NaCl (TD.94045; Table 1), 12 were fed an intermediate salt diet of 4% NaCl (TD.150218; Table 2), 12 were fed a high salt diet of 8% NaCl (TD.150219; Table 3), and 12 were fed a high fructose diet of 64% fructose (TD.06702; Table 4). Rat systolic blood pressure measurements were taken every 3 days for 8 weeks. Systolic blood pressure measurements were recorded due to previous studies indicating that high salt diets could induce systolic blood pressure changes (Walkowska *et al.*, 2015). A minimum of five acceptable blood pressure measurement cycles per rat were averaged; any average with a standard deviation greater than 30 was discarded. Rat weight was also measured every 3 days for 8 weeks. At 8 weeks, rats were euthanized using carbon dioxide, and 14 tissues were harvested per rat. Tissues were harvested in 30 mg samples from the skin (ventral midline), skeletal muscle (pectoralis major), heart (apex), lung, aorta, adipose tissue (abdomen), liver, kidney medulla, kidney cortex, pancreas, stomach, small intestine, brain, and blood. All samples were flash frozen using liquid nitrogen before being stored in an -80 °C freezer until processing.

**Table 1:** Control diet 0.25% NaCl, 6% Fructose: TD.94045 AIN-93G purified diet

Formula			g/kg
Casein			200
L-Cystine			3
Corn Starch			397.486
Maltodextrin			132
Sucrose			100
Soybean Oil			70
Cellulose			50
Mineral Mix, AIN-93G-MX (94046)			35
Vitamin Mix, AIN-93-VX (94047)			10
Choline Bitartrate			2.5
TBHQ, antioxidant			0.014
Nutrient Information			
	% by weight		% kcal from
Protein	17.7		18.8
Carbohydrate	60.1		63.9
Fat	7.2		
Kcal/g		3.8	17.2

(Reeves *et al.*, 1993) Formulated for the growth, pregnancy, and lactational phases of rodents

**Table 2:** 4% NaCl Diet: TD.150218 AIN-93G modification

Formula			g/kg
Casein			200
L-Cystine			3
Corn Starch			359.986
Maltodextrin			132
Sucrose			100
Soybean Oil			70
Cellulose			50
Mineral Mix, AIN-93G-MX (94046)			35
Sodium Chloride			37.4
Vitamin Mix, AIN-93-VX (94047)			10
Choline Bitartrate			2.5
TBHQ, antioxidant			0.014
Blue Food Color			0.1
Nutrient Information			
	% by weight		% kcal from
Protein	17.7		19.5
Carbohydrate	56.7		62.6
Fat	7.2		17.9
	Kcal/g	3.6	

A modification of the AIN-93G purified rodent diet (TD.94045) to increase NaCl to 4% (3.74% added to 0.26% from mineral mix).

**Table 3:** 8% NaCl Diet: TD150219 AIN-93G Modification

Formula			g/kg
Casein			200
L-Cystine			3
Corn Starch			319.986
Maltodextrin			132
Sucrose			100
Soybean Oil			70
Cellulose			50
Mineral Mix, AIN-93G-MX (94046)			35
Sodium Chloride			77.4
Vitamin Mix, AIN-93-VX (94047)			10
Choline Bitartrate			2.5
TBHQ, antioxidant			0.014
Orange Food Color			0.1
Nutrient Information			
	% by weight		% kcal from
Protein	17.7		20.3
Carbohydrate	53.1		61
Fat	7.2		18.6
Kcal/g		3.5	

A modification of the AIN-93G purified rodent diet (TD.94045) to increase NaCl to 8% (7.74% added to 0.26% from mineral mix).

**Table 4:** 63% Fructose Diet: TD.06702 AIN-93G Modification

Formula			g/kg
Casein			200
L-Cystine			3
Fructose			629.336
Soybean Oil			70
Cellulose			50
Mineral Mix, AIN-93G-MX (94046)			35
Vitamin Mix, AIN-93-VX (94047)			10
Choline Bitartrate			2.5
TBHQ, antioxidant			0.014
Green Food color			0.15
Nutrient Information			
	% by weight		% kcal from
Protein	17.7		18
Carbohydrate	64.7		65.6
Fat	7.2		16.4
Kcal/g		3.9	

A modification of the AIN-93G purified rodent diet (TD.94045) to replace primary carbohydrate sources with fructose. The vitamin and mineral mixes still use sucrose as a carrier.

### Tissue processing and real-time PCR

All tissues (excluding blood) were disrupted in liquid nitrogen using a mortar and pestle and homogenized in Buffer RLT (Qiagen, Germantown, MD) using a 20-gauge needle and syringe. Total RNA was purified using the RNeasy Mini Kit (heart, liver, kidney cortex, kidney medulla, small intestine, brain, stomach, pancreas, lung), RNeasy Lipid Tissue Mini Kit (adipose tissue), or RNeasy Fibrous Tissue Mini Kit (skin, skeletal muscle, aorta) (Qiagen). Blood RNA was purified using a separate sample collection and purification technique outlined in the QIAamp RNA Blood Mini Kit protocol (Qiagen). RNA concentrations were quantified using a NanoDrop 2000 (Thermo Electron, Madison, WI). cDNA was prepared using the iScript cDNA Synthesis Kit (Bio-Rad, Hercules, CA). SsoAdvanced Universal SYBR Green SuperMix (Bio-Rad) was used for quantitative real-time PCR amplification of cDNA with primers targeting *NFAT5* (Forward: ACGGACAACAAAGGCAACTC; Reverse: CAGGCTGTACCACTATCTTCA), *AR* (Forward: TCAAGGAGCAGGTGGTGAAG, Reverse: TGCATCCAGGGGGAAATAGT), and *18s* (Forward: CGGCTACCACATCCAAGGAA, Reverse: AGCTGGAATTACCGCGGC). Primers were formulated to identify all isoforms of *NFAT5*. *NFAT5* expression was normalized to *18S* expression using the Delta Delta Ct method [2^(18S-NFAT5)]. *AR* expression was also normalized to 18S expression using the Delta Delta Ct method [2^(18s-AR)].

### Statistical analysis

A Dixon’s Q test was used to identify and discard outliers in gene expression data, and a One-way ANOVA with Tukey HSD post-hoc or Bonferroni and Holm post-hoc test was used to determine significance in gene expression values between each diet group (p < 0.05). Data are presented as means, and error bars represent the standard error.

## Results

**Figure 1:**
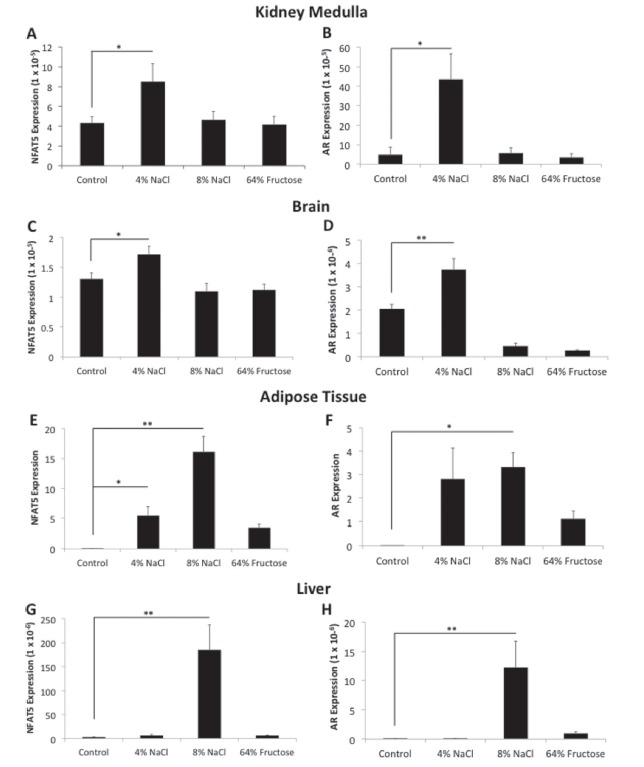
*NFAT5* and *AR* expression are up-regulated in the kidney medulla, brain, adipose tissue, and liver following 8-week administration of a 4% NaCl and/or 8% NaCl diet. Sprague-Dawley rats were fed a control (0.25% NaCl, 6% fructose), 4% NaCl, 8% NaCl, or 64% fructose diet for 8 weeks. Following diet implementation, tissue samples were harvested and flash frozen. RNA was extracted and reverse-transcription was performed to generate cDNA. NFAT5 and AR primers were used to quantify gene expression in the kidney medulla, brain, adipose tissue, and liver. (n = 12-14 rats per diet group,*p < 0.05,*p < 0.01)

**Figure 2:**
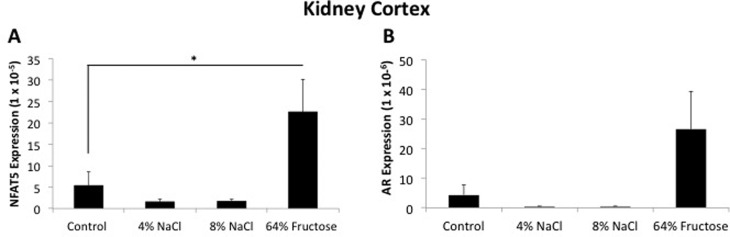
*NFAT5* expression, but not *AR,* is up-regulated in the kidney cortex following 8-week administration of a 64% fructose diet. Sprague-Dawley rats were fed a control (0.25% NaCl, 6% fructose), 4% NaCl, 8% NaCl, or 64% fructose diet for 8 weeks. Following diet implementation, tissue samples were harvested and flash frozen. RNA was extracted and reverse-transcription was performed to generate cDNA. NFAT5 and AR primers were used to quantify gene expression in the kidney cortex. (n = 12-14 rats per diet group,*p < 0.05)

**Figure 3:**
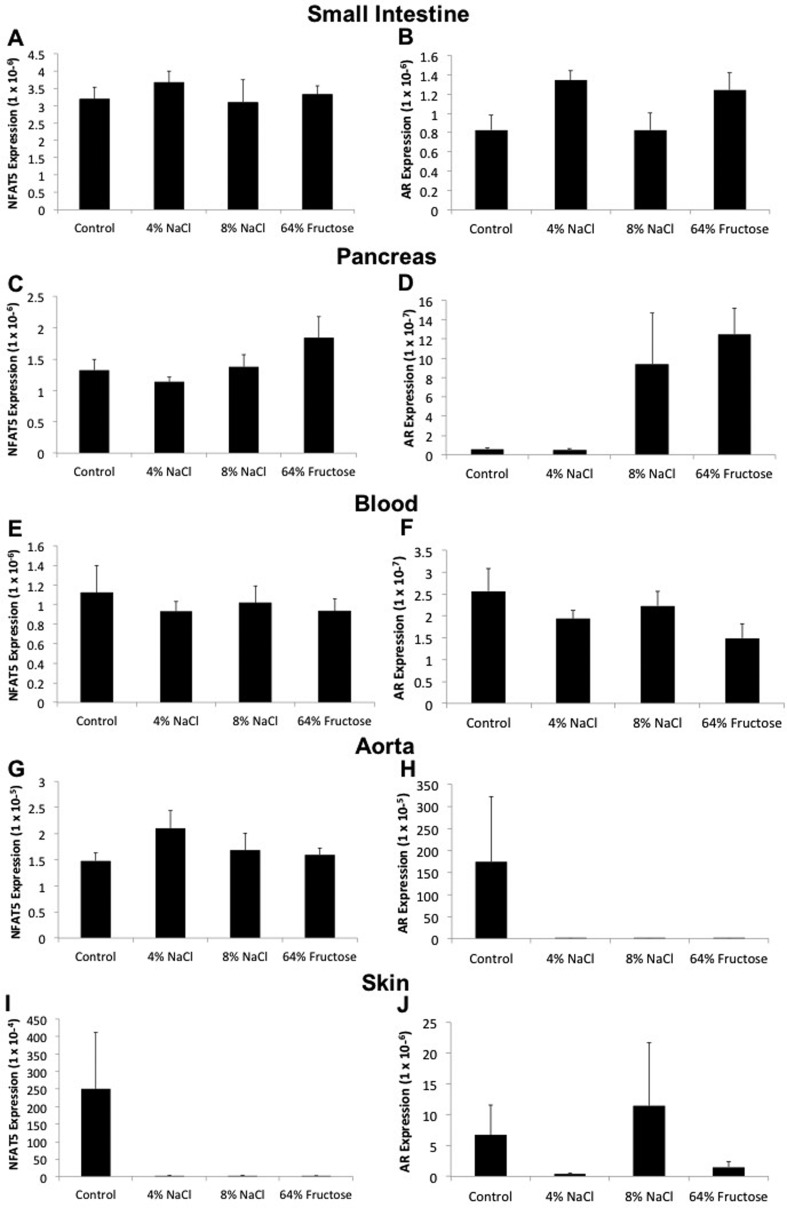
*NFAT5* and *AR* expression remain unchanged in the small intestine, pancreas, blood, aorta, and skin following 8-week administration of a 4% NaCl, 8% NaCl, and/or 64% fructose diet. Sprague-Dawley rats were fed a control (0.25% NaCl, 6% fructose), 4% NaCl, 8% NaCl, or 64% fructose diet for 8 weeks. Following diet implementation, tissue samples were harvested and flash frozen. RNA was extracted and reverse-transcription was performed to generate cDNA. NFAT5 and AR primers were used to quantify gene expression in the small intestine, pancreas, blood, aorta, and skin. (n = 12-14 rats per diet group)

**Figure 4:**
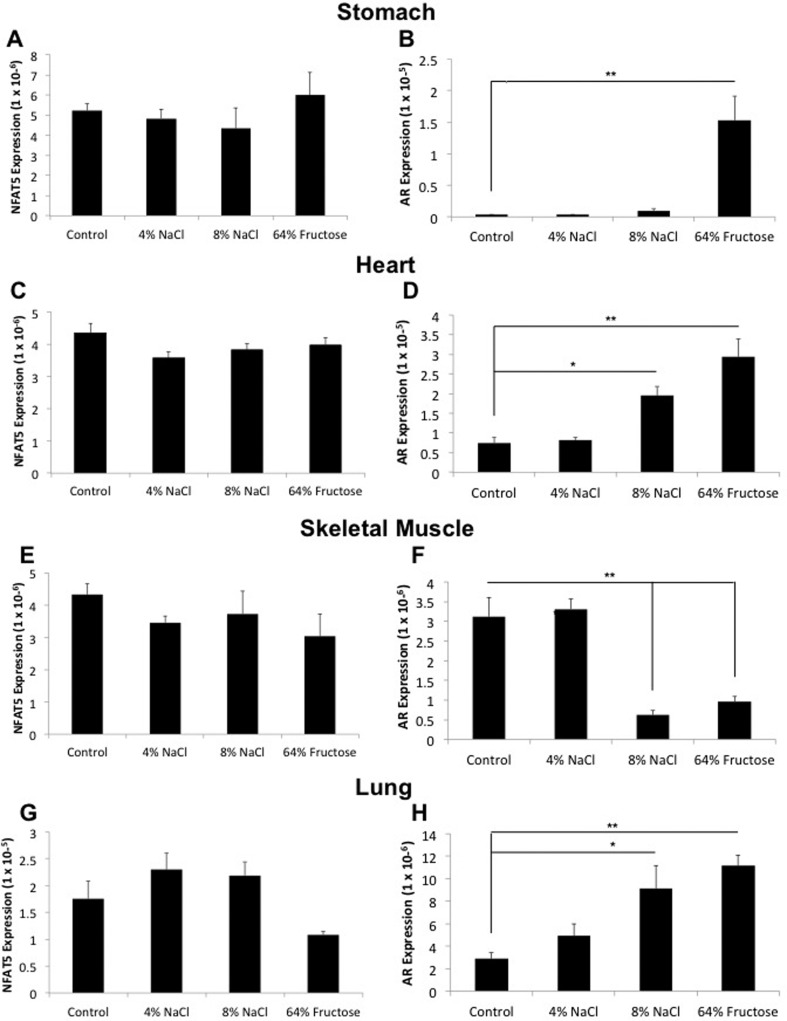
*NFAT5* expression remains unchanged, while *AR* expression is variable in the stomach, heart, skeletal muscle, and lung following 8-week administration of a 4% NaCl, 8% NaCl, and/or 64% fructose diet. Sprague-Dawley rats were fed a control (0.25% NaCl, 6% fructose), 4% NaCl, 8% NaCl, or 64% fructose diet for 8 weeks. Following diet implementation, tissue samples were harvested and flash frozen. RNA was extracted and reverse-transcription was performed to generate cDNA. NFAT5 and AR primers were used to quantify gene expression in the stomach, heart, skeletal muscle, and lung. (n = 12-14 rats per diet group, *p < 0.05,*p < 0.01)

**Figure 5:**
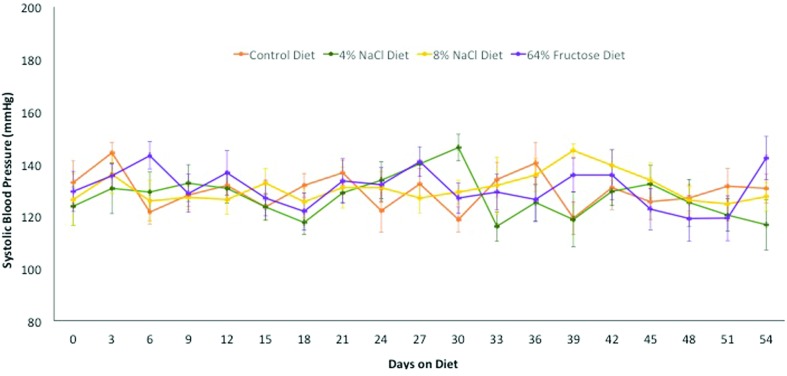
Systolic blood pressure remained constant in Sprague-Dawley rats placed on a 4% NaCl, 8% NaCl, or 64% fructose diet for 8 weeks. Systolic blood pressure was measured using the standard tail cuff method, and averages with a standard deviation greater than 30 were discarded. (n = 12-14 rats per diet group)

**Figure 6:**
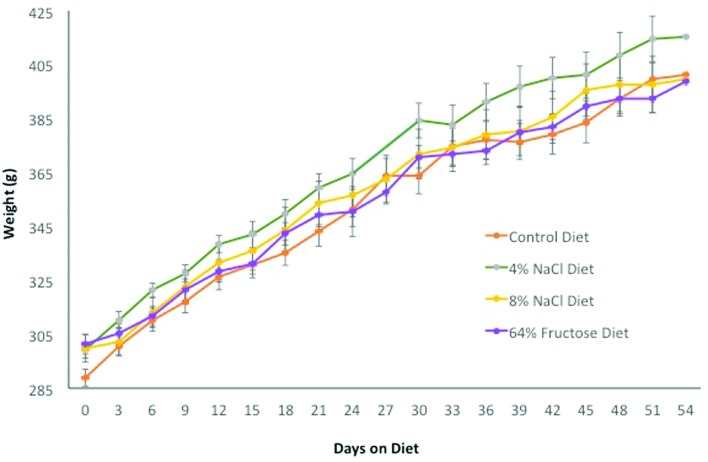
There was no difference in weight between Sprague-Dawley rat diet groups. Rat weights were measured every three days throughout the 8-week diet implementation. (n = 12-14 rats per diet group)

Diet ingredients and nutrient composition percentages for the control diet (Table 1), 4% NaCl diet (Table 2), 8% NaCl diet (Table 3), and 64% fructose diet (Table 4) are provided. *NFAT5* and *AR* were found to be differentially expressed in various tissues of the Sprague-Dawley rat following consumption of a high salt diet and a high fructose diet. *NFAT5* and *AR* gene expression were increased in the kidney medulla, brain, and adipose tissue of rats fed a 4% NaCl diet and in the adipose tissue and liver of rats fed an 8% NaCl diet (Figure 1). The kidney cortex was the only tissue to exhibit an up-regulation of *NFAT5* expression following consumption of a 64% fructose diet (Figure 2). *AR* expression was similarly increased in response to a 64% fructose diet in the kidney cortex, but this data was not significant (p = 0.09) (Figure 2). We observed no change in *NFAT5* or *AR* expression in the small intestine, pancreas, blood, aorta, and skin following all dietary treatments (Figure 3). We similarly observed no change in *NFAT5* expression in the stomach, heart, skeletal muscle, and lung, but these tissues did exhibit variable *AR* expression with the 8% NaCl and 64% fructose diets (Figure 4). Rats fed a 4% NaCl, 8% NaCl, and 64% fructose diet exhibited no change in systolic blood pressure compared to control rats throughout the 8-week diet implementation (Figure 5). There were no observable differences in experimental group rat weights compared to the control group (Figure 6).

## Discussion

NFAT5 is the only transcription factor known to be sensitive to changes in salt-induced osmolarity (Go *et al.*, 2004). Hypertonic stress results in up-regulated NFAT5 RNA levels, translocation of NFAT5 protein to the nucleus, and activation of NFAT5 protein to initiate its binding to the *AR* promoter, amongst others, thus enabling the cell to generate an isosmotic environment (Woo *et al.*, 2002). Our data uniquely show that the kidney medulla, brain, adipose tissue, and liver exhibit increased *NFAT5* and *AR* expression following consumption of a 4% and/or 8% NaCl diet (Figure 1A-H). We herein describe these four tissues as being “salt responsive” due to their exhibiting increased gene expression of the salt-responsive genes *NFAT5* and *AR*.

### Identification of four “salt-responsive” tissues

#### Kidney medulla

*NFAT5* and *AR* expression were increased in the kidney medulla of rats fed a 4% NaCl diet (Figure 1A,B). We hypothesize that increased *NFAT5* and *AR* expression in the kidney medulla is due to the physiological function of this tissue, namely that salt is reabsorbed back into the bloodstream within the kidney medulla. The nephron loop (also called the Loop of Henle) residing within the kidney medulla serves to preserve water and salt through the varied-permeability and osmolarity of the descending and ascending portions of the loop. Salt is concentrated in the descending portion but is then reabsorbed from the ascending portion into the surrounding tissues. We hypothesize that this physiological process creates a hyperosmotic environment within the kidney medulla, which thereby triggers activation of *NFAT5* and *AR* expression to prevent cellular damage. Unexpectedly, we only observed an increase in *NFAT5* and *AR* expression with the 4% NaCl diet and not the 8% NaCl diet. We hypothesize that with the 8% NaCl diet, there may be such an excess of salt present that the kidney prioritizes conservation of water over salt, with more salt being excreted from the body; therefore, a hypertonic environment may not be generated in the kidney medulla under these conditions. Anecdotally, it was observed that rats in the 8% NaCl diet group, compared to the 4% NaCl diet group, drank more water during the diet implementation; however, water consumption was not quantitatively measured in this study.

The role of osmoregulation within the kidney has been well documented due to the aforementioned physiological role renal medulla cells play. Due to the high osmolality that is necessary for the medulla to perform its proper function, genes that enable cells to adapt to these conditions are vital to the survival of the organ and have been identified as key players in the prevention of ischemic kidney injury (Hao *et al.*, 2014). The production of sorbitol via AR has consistently been documented as one of the more significant physiological functions in osmoadaptation in various parts of the kidney. Grunewald *et al.* (2001) observed that AR played a role in osmoregulation throughout all portions of the kidney medulla, indicating a uniform dispersion of expression within the tissue. In dehydration and water conservation studies, *NFAT5* and *AR* expression were increased in the kidneys of dehydrated rats and also in the kidneys of wood frogs during anoxia, when water conservation is essential for survival (Cha *et al.*, 2001; Al-Attar *et al.*, 2017). These studies highlight the role of NFAT5 and AR in cellular protection and water conservation, whereas our study provides evidence of the significance of osmoregulatory gene expression within the renal medulla following high salt diet consumption.

#### Brain

*NFAT5* and *AR* expression were increased in the brains of rats fed a 4% NaCl diet (Figure 1C,D). Maallem *et al.* (2008) showed that intraperitoneal injection of a hypertonic sucrose solution induced NFAT5 protein expression in the rat brain, suggesting that high plasma osmolality can activate NFAT5 translation within neurons and/or glial cells, despite the presence of the blood brain barrier. Zhang *et al.* (2015) further showed that when mice are fed a high salt diet and experience ischemic events, the blood brain barrier can become disrupted due to tight junction protein loss, which results in leukocyte infiltration into the brain. Since rats fed an 8% NaCl diet in our study did not exhibit elevated *NFAT5* expression levels in the brain, but rats on a 4% NaCl diet did, it is possible that hypertonicity is not activating *NFAT5* expression under 4% NaCl dietary conditions, but rather, tonicity-independent mechanisms may be activating *NFAT5* expression. One such tonicity-independent mechanism was identified by Jeong *et al.* (2016) whereby *NFAT5* expression was enhanced in microglia in response to LPS, interferon-gamma, and interleukin 4 inflammatory cytokine stimulation. Since high salt diets can enhance inflammatory cytokine release, it is perhaps through this tonicity-independent mechanism that *NFAT5* expression is indirectly being altered in the 4% NaCl diet group (Hucke *et al.*, 2016).

#### Adipose tissue

*NFAT5* expression was increased in the adipose tissue of rats fed a 4% NaCl and 8% NaCl diet, and *AR* expression was increased in those fed an 8% NaCl diet (Figure 1E,F). While currently unknown, there may be a number of factors causing an increase in *NFAT5* and *AR* expression in this tissue. Adipose tissue could act as a collecting source for salt, similar to how small pockets of hypertonicity can develop within the skin (Machnik *et al.*, 2009). High salt diets could similarly be activating NFAT5 within macrophages present in adipose tissue, as macrophages are known to be sensitive to changes in salt concentrations (Jantsch *et al.*, 2014).

#### Liver

*NFAT5* and *AR* expression were increased in the livers of rats fed an 8% NaCl diet (Figure 1G,H). As the liver receives blood directly from the intestines, and this blood contains all of the nutrients that are absorbed from food, the liver must be able to adapt to varying levels of osmolytes present in hepatic blood throughout the day. The 4% NaCl diet did not alter *NFAT5* or *AR* expression, suggesting that either a hypertonic environment is not created in the liver with slightly elevated salt in the diet or that the liver is only sensitive to large increases in dietary salt (*i.e.* 8% NaCl). In 8% NaCl-fed rat livers, *NFAT5* expression was increased 55-fold, compared to more modest increases in *NFAT5* in the kidney medulla, brain, and adipose tissue under 4% NaCl and 8% NaCl diet conditions. This suggests that NFAT5 plays a critical role in modulating the osmotic environment in the liver following high salt consumption. NFAT5 osmoadaptation has been linked to the prevention of inflammatory diseases of the liver caused by the pathologically hypertonic environment of the organ; this provides further insight into the role of NFAT5 in damage prevention under hyperosmotic conditions within the liver (Neuhofer, 2010).

### The first evidence of a high fructose diet altering *NFAT5* expression

*NFAT5* expression was increased in the kidney cortex of rats fed a 64% fructose diet. *AR* expression was similarly increased in the 64% fructose diet condition in the kidney cortex, but this data was not significant (p = 0.09) (Figure 2A,B). The proximal convoluted tubule of the nephron is located in the kidney cortex, and it is here where sugars are reabsorbed from the nephron into the extracellular space to then be reabsorbed back into the bloodstream. We hypothesize that movement of sugar out of the proximal convoluted tubule in rats placed on a high fructose diet would result in hyperosmotic conditions in the extracellular environment of the kidney cortex. It is therefore possible that *NFAT5* expression is up-regulated in the kidney cortex to assist cells in adapting to the hyperosmotic environment generated by increased extracellular fructose concentrations. Although *AR* expression was not significantly up-regulated in the kidney cortex of rats fed a 64% fructose diet, it was elevated, and significance could be reached with a slightly larger sample size. Of note, *AR* expression was not increased when human renal proximal tubule cells were exposed to increased glucose levels *in vitro*, unlike that of hyperosmotic conditions induced by increased salt in previous studies (Bondy *et al.*, 1989; Petrash *et al.*, 1992).

### Tissues experiencing no alteration in NFAT5 or AR expression on all diets

We observed no change in *NFAT5* or *AR* expression in the small intestine, pancreas, blood, aorta, and skin following all dietary treatments (Figure 3). Previous studies found an increase in plasma osmolarity under acute increased salt diets but have been inconsistent with overall measurements over extended high salt diets (de Wardener *et al.*, 2004). Therefore, we did not quantify the osmolarity of these tissues, since vapor pressure osmometer measurements can often be inaccurate and misleading; however, this information could shed light as to why these select tissues showed no alteration in *NFAT5* or *AR* expression (Stahl *et al.*, 2007). Although high levels of salt and/or fructose would be traveling through the small intestine in each rat diet group, our data indicate that this did not affect *NFAT5* or *AR* expression (Figure 3A, B). This could either be due to the relatively short amount of time in which food is present in the small intestine or to a lower sensitivity of the intestinal tissue to changes in tonicity. The pancreas does not directly come into contact with high salt or high fructose concentrations in the intestines, but rather plays an accessory role by injecting enzymes into the small intestine to aid in digestion; therefore, it is reasonable that the pancreas would most likely not experience alterations in tonicity to affect *NFAT5* and *AR* expression (Figure 3C,D). Although Machnik *et al.* (2009) demonstrated that mice consuming a high salt diet develop local hypertonic microenvironments within the skin, and this local hypertonicity drives an increase in NFAT5 expression and activity, skin tissue harvested in this study did not show an increase in *NFAT5* or *AR* expression. Our results show a large degree of error in *NFAT5* and *AR* expression within the skin of rats from multiple diet groups; this could represent the variability with which salt can accumulate in different portions of the skin (Figure 3I,J).

### Alterations in *AR* expression independent of *NFAT5*

Although *NFAT5* expression was not altered in the stomach, heart, skeletal muscle and lung, these tissues did exhibit variable *AR* expression with the 8% NaCl and 64% fructose diets (Figure 4A-H). It is possible that: 1) NFAT5 protein is binding to the *AR* promoter to activate AR transcription independent of an increase in NFAT5 RNA, 2) *AR* is being up-regulated via an NFAT5-independent mechanism in response to consumption of an 8% NaCl or 64% fructose diet in these tissues, or 3) we simply were not able to measure the change in *NFAT5* expression in these tissues. Maallem *et al.* (2006) similarly showed an increase in AR expression independent of an increase in NFAT5 expression in rat brains placed under hyperosmotic conditions.

### Use of the rat model

Rats fed a 4% NaCl, 8% NaCl, and 64% fructose diet exhibited no change in systolic blood pressure throughout the 8-week implementation of the diet (Figure 5). Sprague-Dawley rats were utilized in these studies due to previous literature indicating that this rat strain develops hypertension within 6 weeks of eating an 8% NaCl diet and within 2 weeks of consuming a 64% fructose diet (Hwang *et al.*, 1987; Catena *et al.*, 2003; Gu *et al.*, 2008). Contrary to these studies, we did not measure a diet-induced alteration in blood pressure in any of our experimental diet groups after 8 weeks of the diet. It is possible that genetic drift has occurred in the Sprague-Dawley rat since the prior studies by Gu *et al.* (2008), Hwang *et al.* (1987), and *Catena et al.* (2003), or that slightly different Sprague-Dawley genetic strains are bred at different laboratories. Of note, our Sprague-Dawley rats exhibited a higher systolic blood pressure (ranging from 125-135 mmHg) at the start of our study, compared to a lower systolic blood pressure (ranging from 120-125 mmHg) measured at the start of the study conducted by Gu *et al.* (2008). Therefore, it is important to highlight that the Sprague-Dawley rat may no longer be an optimal model for short-term high salt and high fructose diet-induced hypertension studies. In humans, there is clear evidence that not all high salt diet regimens generate increases in blood pressure; however, these diets can alter the structure and function of the cardiovascular system, kidneys, and brain (Farquhar *et al.*, 2015). Therefore, it is important to understand how these high salt diets alter gene expression independent of blood pressure changes. Weight gain was not observed in the rats over the 8-week diet. A previous study found that prolonged high fructose diets, greater than 6 months, induced leptin resistance prior to body weight increases (Shapiro *et al.*, 2008). The rats in this study were likely not fed the high fructose diet long enough to induce leptin resistance and subsequent weight gain.

### Conclusion and future directions

We show that two hypertonicity-sensitive genes, *NFAT5* and *AR*, are differentially expressed in various tissues of the body. We characterize the kidney medulla, liver, brain, and adipose tissue as being “salt-responsive” tissues exhibiting upregulated *NFAT5* and *AR* expression and uncover that a high fructose diet can lead to enhanced *NFAT5* expression in the kidney cortex. We hypothesize that NFAT5 plays an osmoprotective role in these “salt-responsive” tissues; however, over time, high salt diet feeding may result in NFAT5-driven expression of pro-inflammatory cytokines, leading to potential cellular toxicity and tissue damage. Although not in the scope of this study, beneficial future studies would entail determining how diet alters NFAT5 protein expression and translocation into the nucleus and NFAT5 binding activity on various gene promoters. It would additionally be beneficial to examine the effects of fructose on NFAT5 regulation *in vitro*, including elucidating the molecular mechanisms involved in the potential fructose-mediated activation of *NFAT5* gene expression. A deeper examination of the cell signaling mechanisms involved in dietary induction of NFAT5 and AR, as well as other NFAT5-dependent genes, would provide a larger picture of the mechanisms through which tissues in the body respond to consumption of high salt and high fructose diets.
